# NADPH Oxidase RbohD and Ethylene Signaling are Involved in Modulating Seedling Growth and Survival Under Submergence Stress

**DOI:** 10.3390/plants9040471

**Published:** 2020-04-08

**Authors:** Chen-Pu Hong, Mao-Chang Wang, Chin-Ying Yang

**Affiliations:** 1 Department of Agronomy, National Chung Hsing University, Taichung 40227, Taiwan; xo7961@hotmail.com; 2 Department of Accounting, Chinese Culture University, Taipei 11114, Taiwan; wmaochang@yahoo.com.tw

**Keywords:** ethylene, Ein2, germination, RbohD, submergence, hypoxia

## Abstract

In higher plants under low oxygen or hypoxic conditions, the phytohormone ethylene and hydrogen peroxide (H_2_O_2_) are involved in complex regulatory mechanisms in hypoxia signaling pathways. The respiratory burst oxidase homolog D (RbohD), an NADPH oxidase, is involved in the primary stages of hypoxia signaling, modulating the expression of downstream hypoxia-inducible genes under hypoxic stress. In this study, our data revealed that under normoxic conditions, seed germination was delayed in the *rbohD*/*ein2-5* double mutant, whereas postgermination stage root growth was promoted. Under submergence, the *rbohD/ein2-5* double mutant line had an inhibited root growth phenotype. Furthermore, chlorophyll content and leaf survival were reduced in the *rbohD/ein2-5* double mutant compared with wild-type plants under submerged conditions. In quantitative RT-PCR analysis, the induction of *Ethylene-responsive factor 73*/*hypoxia responsive 1* (*AtERF73/HRE1*) and *alcohol dehydrogenase 1* (*AtADH1*) transcripts was lower in the *rbohD/ein2-5* double mutant during hypoxic stress than in wild-type plants and in *rbohD* and *ein2-5* mutant lines. Taken together, our results indicate that an interplay of ethylene and RbohD is involved in regulating seed germination and post-germination stages under normoxic conditions. Moreover, ethylene and RbohD are involved in modulating seedling root growth, leaf chlorophyll content, and hypoxia-inducible gene expression under hypoxic conditions.

## 1. Introduction

Climate change—induced flooding is a major global natural disaster. Flooding and heavy rain can cause soil compaction, reducing soil oxygen concentration and resulting in hypoxic stress—induced plant damage. The plant hormone ethylene participates in regulating stress-inducible genes to help plants to adapt to various environmental stresses, especially in hypoxia signaling caused by flooding [1,2,3].

Ethylene controls diverse physiological pathways involved in plant growth and developmental processes including the regulation of seed germination and leaf senescence and the promotion of pollen tube growth and fruit ripening [4,5,6]. Ethylene also contributes to plant responses to different biotic and abiotic stresses such as insect or microbial infections, drought, and salt conditions [7,8,9]. Several studies have shown that the ER-located membrane protein ETHYLENE INSENSITIVE 2 (EIN2) acts as a key signal transducer that positively signals downstream to members of the ETHYLENE INSENSITIVE 3 (EIN3) family of transcription factors located in the nucleus. EIN3 further binds to the promoters of ethylene-response genes, activating their expression in an ethylene-dependent manner to activate downstream ethylene responses [10,11].

Under oxygen deficient conditions, ethylene plays a major role in the regulation of abscisic acid (ABA) and gibberellic acid (GA) to influence cell elongation [12]. In addition to cell elongation, ethylene-induced programmed cell death through reactive oxygen species (ROS) production leads to the formation of aerenchyma and emergence of adventitious roots in maize and rice [13,14,15]. Hydrogen peroxide (H_2_O_2_) acts as a secondary messenger downstream of the ethylene signal to promote aerenchyma formation under oxygen-deficient conditions [16]. H_2_O_2_ is a type of ROS. ROS include H_2_O_2_, superoxide anion radicals (O_2_^−^), hydroxyl radicals (OH), and singlet oxygen (^1^ O_2_), all of which are byproducts of aerobic metabolism [17]. NADPH oxidases, also named respiratory burst oxidase homologs (RBOHs), catalyze superoxide radical generation in plant apoplasts. Subsequently, superoxide radicals are converted into H_2_O_2_ by the activity of cell wall-localized antioxidant enzymes termed superoxide dismutases (SODs). H_2_O_2_ regulates the induction of *ethylene-responsive factor 73* (*ERF73*) and *alcohol dehydrogenase 1* (*ADH1*) expression under oxygen deprivation [18,19]. In *Arabidopsis thaliana*, RBOHs are members of a multigene family composed of 10 RBOH genes (AtRBOH A-J) that participate in ROS production in response to environmental stresses [20].

Plant RBOHs display different expression patterns during developmental processes and in response to various abiotic stresses and biotic interactions, either pathogenic or symbiotic [21]. AtRBOHC was revealed to be activated in Ca_2_^+^-dependent signaling triggered by the mechanical stimulation of root hairs [22]. Mild salt stress reportedly increases AtRbohD transcript levels, and AtRbohD and AtRbohF are involved in the ABA signaling network in guard cells [23,24]. AtRbohD mediates rapid systemic signaling in response to wounding, heat, cold, high-intensity light, and salinity stresses [25].

Our previous studies revealed that the accumulation of H_2_O_2_ was reduced in *ein2-5* and *rbohD*-knockout (*rbohD*-ko) mutants during hypoxic stress. The induction of hypoxia-inducible genes was also reduced in *rbohD*-ko mutants under hypoxic stress. AtRbohD plays a major role in the early stages of the stress response to oxygen deprivation [19,26]. Although AtRbohD is involved in hypoxia signaling, little is known about the relationship between ethylene and RbohD in submergence stress. In this study, to clarify the functional relationship between ethylene and H_2_O_2_ in the hypoxia signaling pathway, we analyzed *rbohD/ein2-5* double mutant and wild-type plants under hypoxic stress. Our results demonstrate that the interplay between ethylene and H_2_O_2_ is involved in modulating seed germination, seedling root growth, leaf chlorophyll content, and hypoxia-inducible gene expression under hypoxic conditions.

## 2. Results

### 2.1. The rbohd/ein2-5 Double Mutant Exhibited Ethylene Insensitive, Delayed Seed Germination and Increased Postgermination Root Growth

**Figure 1:**
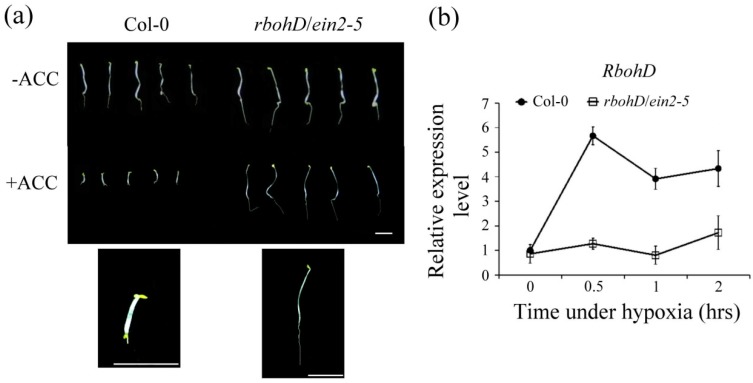
Phenotypes of wild-type Col-0 and *rbohD*/*ein2-5* double mutants in *Arabidopsis*. (**a**) The wild-type (Col-0) and double mutant (*rbohD*/*ein2-5*) seeds were grown on 1/2 MS medium with 5 μM ACC or without ACC for 4 d under dark conditions. Bar = 0.5 cm. (**b**) Quantitative RT-PCR analyses of transcript levels of *RbohD* in *Arabidopsis* Col-0 and *rbohD*/*ein2-5* double mutants in response to hypoxic stress for 0, 0.5, 1, and 2 h. Total RNAs were isolated from roots of 14-day-old seedlings after hypoxia treatment at indicated times and levels of *RbohD* mRNA were determined. Relative transcript amounts were calculated and normalized to *Actin* mRNA levels. Values represent means ± SD from five biologically independent experiments.

The induction of H_2_O_2_ and levels of *AtERF73/HRE1* and *ADH1* are reduced in *rbohD* and *ein2-5* single mutants under hypoxic stress [19,26]. To further investigate the interplay of ethylene and H_2_O_2_ signaling during hypoxia, we obtained *rbohD/ein2-5* double mutant lines by crossing single *rbohD* and *ein2-5* homozygous mutants. After seeds were grown on 1/2 strength Murashige and Skoog (MS) medium with or without 1-aminocyclopropane-1-carboxylic acid (ACC) treatment for 4 d in dark conditions, the etiolated seedlings of the *rbohD/ein2-5* double mutant line did not exhibit the triple response (Figure 1a). Increased *RbohD* transcript levels were not induced in the *rbohD/ein2-5* double mutant line after hypoxic treatment (Figure 1b).

**Figure 2:**
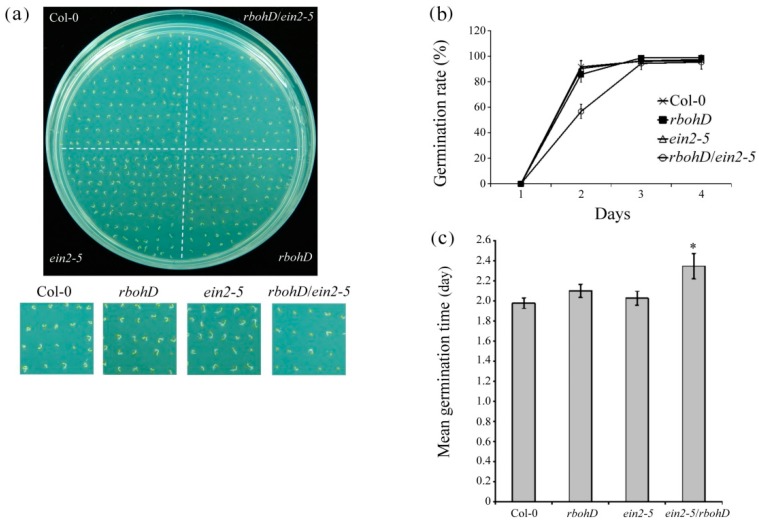
Germination assay of wild-type, *rbohD*, *ein2-5* and *rbohD*/*ein2-5* mutants. (**a**) Germination of wild-type, *rbohD* and *ein2-5* single mutants, and double mutant *rbohD*/*ein2-5* seeds. Seeds were sown in 1/2 MS medium and grown for 3 d at 4 °C in dark conditions; photographs were taken after 2 d. (**b**,**c**) Germination rates and mean germination times of wild-type, *rbohD* and *ein2-5* single mutants, and double mutant *rbohD*/*ein2-5* seeds. Seeds were scored for 1, 2, 3, and 4 d after sowing from at least 100 seeds of each genotype with three replicates each. Error bars represent SD. * *p* < 0.05, versus wild-type (Student’s *t* test).

The *rbohD/ein2-5* double mutant seeds germinated more slowly than wild-type, *rbohD*, and *ein2-5* single mutants under normaxic conditions; 90%, 85%, and 91% of wild-type, *rbohD*, and *ein2-5* single mutant seeds germinated after 2 d, whereas only 56% of *rbohD/ein2-5* double mutant seeds germinated. Germination was largely completed after 3 d incubation (Figure 2a,b). The mean germination time of seeds from wild-type, *rbohD* and *ein2-5* single mutants, and that from the *rbohD/ein2-5* double mutant, was 1.98, 2.10, 2.03, and 2.35 d, respectively (Figure 2c).

**Figure 3:**
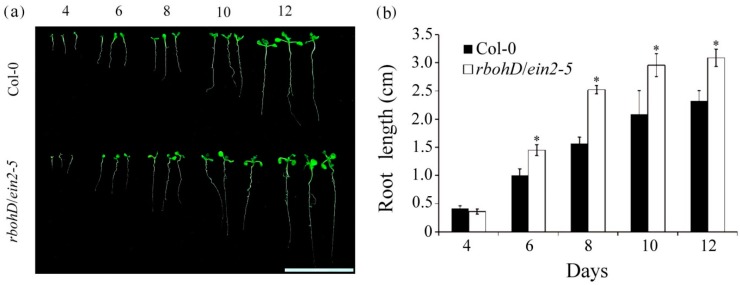
Comparison of root growth in wild-type and *rbohD*/*ein2-5* double mutant seedlings. (**a**) Phenotypes of seedlings grown on 1/2 MS medium for 4, 6, 8, 10, and 12 d. Bar = 2 cm. (**b**) Average root length of seedlings grown on 1/2 MS. Error bars represent SD of 100 seedlings of each genotype, obtained from three biologically independent experiments. * *p* < 0.05, versus wild-type (Student’s *t* test).

To determine whether ethylene and H_2_O_2_ signaling affected postgermination seedling development, the *rbohD/ein2-5* double mutant seedlings were grown for 4, 6, 8, 10, and 12 d. The root lengths of the *rbohD/ein2-5* double mutant lines were significantly longer than those of the wild-type seedlings (Figure 3a). When grown for 4, 6, 8, 10, and 12 d, wild-type seedlings developed roots with average lengths of 0.42, 1.00, 1.57, 2.09, and 2.32 cm, respectively, whereas the average root length of *rbohD/ein2-5* double mutants was 0.36, 1.45, 2.52, 2.95, and 3.09 cm, respectively (Figure 3b). These results indicate that in the *rbohD/ein2-5* double mutant, seed germination was delayed and the postgermination root growth rate increased.

### 2.2. The rbohD/ein2-5 Double Mutant Line Exhibited no Inhibition of Root Growth Following Submergence

**Figure 4:**
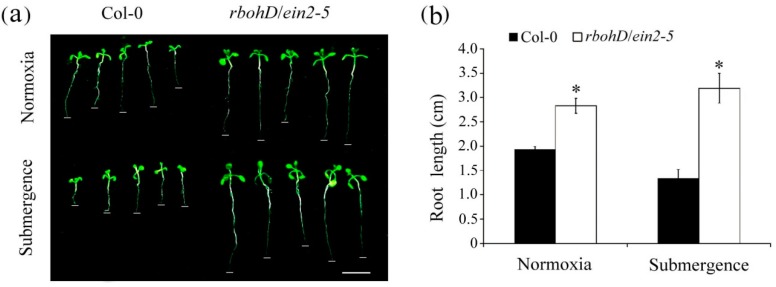
Comparison of root growth in wild-type and *rbohD*/*ein2-5* double mutant seedlings after submergence. (**a**) Phenotypes of seedlings grown on 1/2 MS medium for 14 d and then treated with submerged conditions for an additional 7 d. Bar = 1 cm. (**b**) Average root length of seedlings grown under normaxic condition and submerged conditions. Error bars represent SD for at least 100 seedlings of each genotype, obtained from three biologically independent experiments. * *p* < 0.05, versus wild-type plants (Student’s *t* test).

Ethylene plays a role in inhibiting root length during plant developmental processes. Hydrogen peroxide also functions in inhibiting root length [27,28]. To determine whether ethylene signaling and H_2_O_2_ affect root growth in response to submergence, 7-day-old wild-type and *rbohD/ein2-5* double mutant seedlings were treated with normoxic or submerged conditions for 7 d. Root lengths were significantly reduced in wild-type plants compared with the *rbohD/ein2-5* double mutant lines (Figure 4a). The average root lengths of wild-type and the *rbohD/ein2-5* double mutant under the normaxic condition were 1.94 and 2.83 cm, respectively, whereas the average root lengths of wild-type and the *rbohD/ein2-5* double mutant under submergence were 1.34 and 3.19 cm, respectively (Figure 4b). The root lengths of the *rbohD/ein2-5* double mutant increased by 113% under submerged conditions compared with under the normaxic condition, whereas those of wild-type plants decreased by 69%. These results indicate that root growth was not inhibited in submerged *rbohD/ein2-5* double mutant lines.

### 2.3. Reduced Chlorophyll Content and Survival of rbohD/ein2-5 Double Mutant Plants under Submerged Conditions

**Figure 5:**
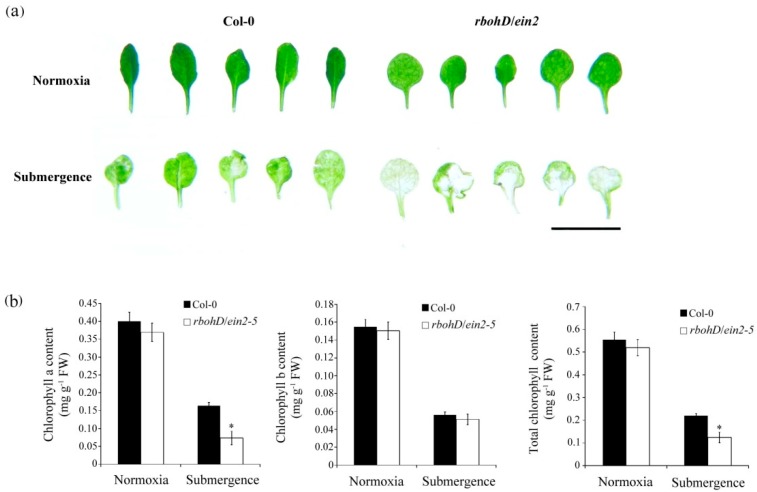
Chlorophyll content of wild-type plants and *rbohD*/*ein2-5* double mutants after submergence stress. (**a**) Phenotype of 7-day-old wild-type plants (Col-0) and double mutant *rbohD*/*ein2-5* leaves after treatment under normoxic conditions and submerged conditions for 12 d. Bar = 1 cm. (**b**) Contents of chlorophyll a, b, and total chlorophyll of 7-day-old seedlings after treatment under normoxic conditions and submerged conditions for 12 d. Values represent means ± SD from three biologically independent experiments. * *p* < 0.05, versus wild-type (Student’s *t* test).

**Figure 6:**
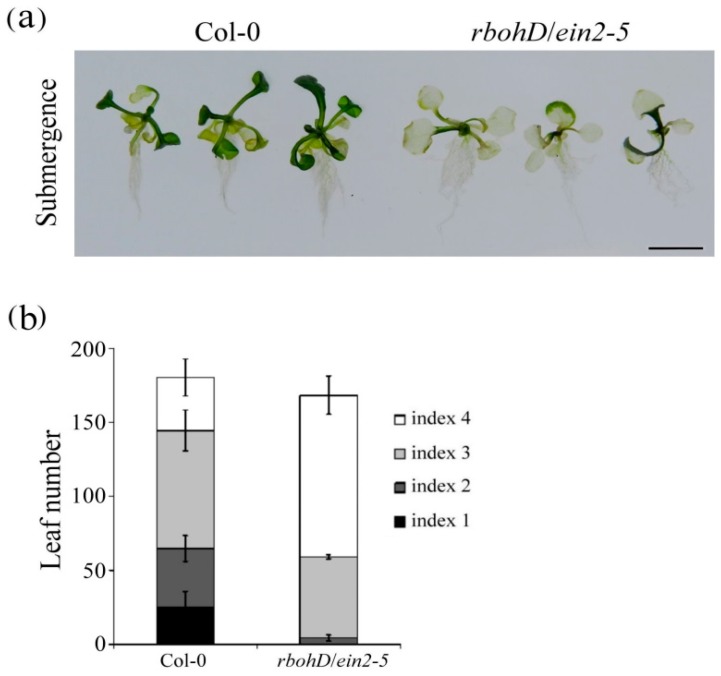
Sensitivity of the *rbohD*/*ein2-5* double mutant to submerged conditions. (**a**) Phenotypes of 7-day-old wild-type plants and *rbohD*/*ein2-5* double mutant seedlings after 12 d of submergence and 5 d of subsequent recovery. Bar = 1 cm. The photograph provides the results for three independent seedlings. (**b**) Quantification of the phenotype. Levels of damage were defined using an index based on the percentage of chlorotic leaves. Index 1 = 100% green leaves, Index 2 = over 50% green leaves, Index 3 = less than 50% green leaves, and Index 4 = 0% green leaves. The same results were obtained from three independent experiments (*n* ≥ 25).

To further assess the role of ethylene and H_2_O_2_ signaling under submerged conditions, chlorophyll content was determined during submergence. The 7-day-old wild-type and *rbohD/ein2-5* double mutant seedlings were submerged for 12 d. The *rbohD/ein2-5* double mutant exhibited more severe leaf etiolation than the wild-type plants did under submergence (Figure 5a). Contents of chlorophyll a and total chlorophyll were significantly decreased in the *rbohD/ein2-5* double mutant line after submergence treatment (Figure 5b). This effect was also observed in 7-day-old seedlings submerged for 12 d then left to recover for 5 d. The degree of leaf damage following submergence of the etiolated seedlings was analyzed and classified according to an index scale ranging from 1 to 4, where index scores of 1 and 2 represent 100% and over 50% green leaf area, respectively, and scores 3 and 4 represent less than 50% and 0% green leaf area, respectively. These data indicate that more than 95% of the *rbohD/ein2-5* double mutant seedlings exhibited a phenotype with leaf damage (Figure 6a,b), suggesting that ethylene and H_2_O_2_ signaling positively regulate aboveground leaf survival under submergence stress.

### 2.4. Effects of the rbohD/ein2-5 Double Mutant Line on Hypoxia-Inducible Genes under Hypoxic Stress

**Figure 7:**
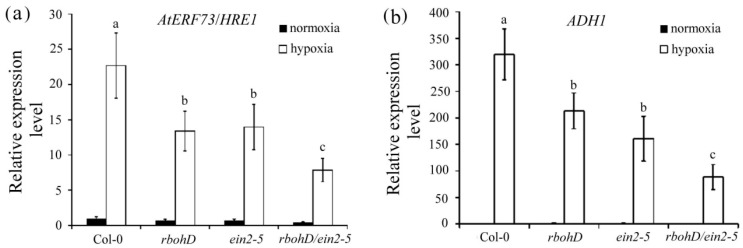
Transcript levels of hypoxia-inducible genes encoding AtERF73/HRE1 and ADH1 in *rbohD*/*ein2-5* lines after hypoxia treatment. Total RNAs were isolated from roots of 14-day-old Col-0, *rbohD*, *ein2-5*, and *rbohD*/*ein2-5* seedlings after 3 h of hypoxia treatment. Quantitative RT-PCR analyses of transcript levels of *AtERF73/HRE1* (**a**) and *ADH1* (**b**) genes. Values are means ± SD from three biologically independent experiments (*n* = 3). Values with different letters are significantly different at *p* < 0.05, according to a post-hoc LSD test.

To determine how ethylene and H_2_O_2_ signaling affects hypoxia responses, we examined the expression of *AtERF73/HRE1* and *ADH1* in wild-type, *rbohD* and *ein2-5* single mutants, and the *rbohD/ein2-5* double mutant using qRT-PCR. The results indicate that under hypoxic conditions, the induction of *AtERF73/HRE1* and *ADH1* mRNA was significantly reduced in *rbohD* and *ein2-5* single mutants and in the *rbohD/ein2-5* double mutant lines compared with wild-type plants (Figure 7a,b). The *rbohD/ein2-5* double mutant had the most pronounced reduction in hypoxia-induced transcription. Taken together, these results suggest that ethylene and H_2_O_2_ signaling synergistically regulated hypoxia-inducible gene responses.

## 3. Discussion

Plants are sessile organisms that have evolved integrated complex regulation systems to respond to stress. Oxygen deprivation is a common abiotic stress often associated with flooding. It affects plant growth, development, and survival. The role of ethylene in oxygen sensing and signaling is complex [29]. Recent studies have demonstrated that when submergence is established, gas diffusion of ethylene is restricted, and it is trapped in the plant, triggering its signaling pathway at early submergence. When submergence is prolonged, ethylene synthesis is reduced due to hypoxia; group VII ethylene response factor (ERFVII) activity is activated by hypoxia signaling [30,31,32]. In our previous reports, we demonstrated that the NADPH oxidase RbohD plays a major role in the early stages of hypoxic stress responses. Ethylene signaling modulates H_2_O_2_ signaling by regulating the expression of Rboh genes [19,26]. RbohD triggers cell death following fungal infection, and crosstalk between the salicylic acid and ethylene signaling pathways inhibits death in neighboring cells [33]. Furthermore, the double null mutant *atrbohD*/*F* is more sensitive to oxygen deficiency than wild-type plants and single mutants *atrbohD* and *atrbohF* are [34]. Little is known about the ethylene-mediated regulation of RbohD in the hypoxia signaling pathway.

The present study revealed that *rbohD/ein2-5* double mutants had delayed seed germination but increased root growth compared with wild-type plants (Figure 2 and Figure 3). During seed germination, endogenous ethylene promotes seed germination by decreasing sensitivity to endogenous ABA [35]. H_2_O_2_, acting as a signaling molecule, mediates seed dormancy breaking and germination through the upregulation of ABA catabolism and GA biosynthesis [36]. Our data demonstrate that the mean seed germination time was significantly reduced in wild-type plants and the *rbohD* and *ein2-5* single mutants compared with the *rbohD/ein2-5* double mutant (Figure 2c), indicating that the interplay between ethylene signaling and H_2_O_2_ signaling modulates seed germination. Previously, we determined that under normoxic conditions, postgermination seedling root lengths of wild-type plants and *rbohD* single mutants do not differ [26]. Notably, the current study revealed that the *rbohD/ein2-5* double mutant had faster root growth 6–12 d after germination under both normoxic (Figure 3b) and submerged (Figure 4) conditions. Ethylene has been demonstrated to inhibit root cell elongation by upregulating auxin biosynthesis in Arabidopsis seedlings; accumulated H_2_O_2_ also inhibits root cell elongation and root growth [37,38,39]. Under submerged conditions, root length is reduced by oxygen deficiency [40]. However, the *rbohD/ein2-5* double mutant did not display inhibited root growth under submergence; instead, it had longer roots than wild-type plants did. These results suggest that crosstalk between ethylene signaling and H_2_O_2_ signaling, in addition to delaying seed germination, affects the root growth rate of postgermination seedlings under both normoxic and hypoxic conditions.

**Figure 8:**
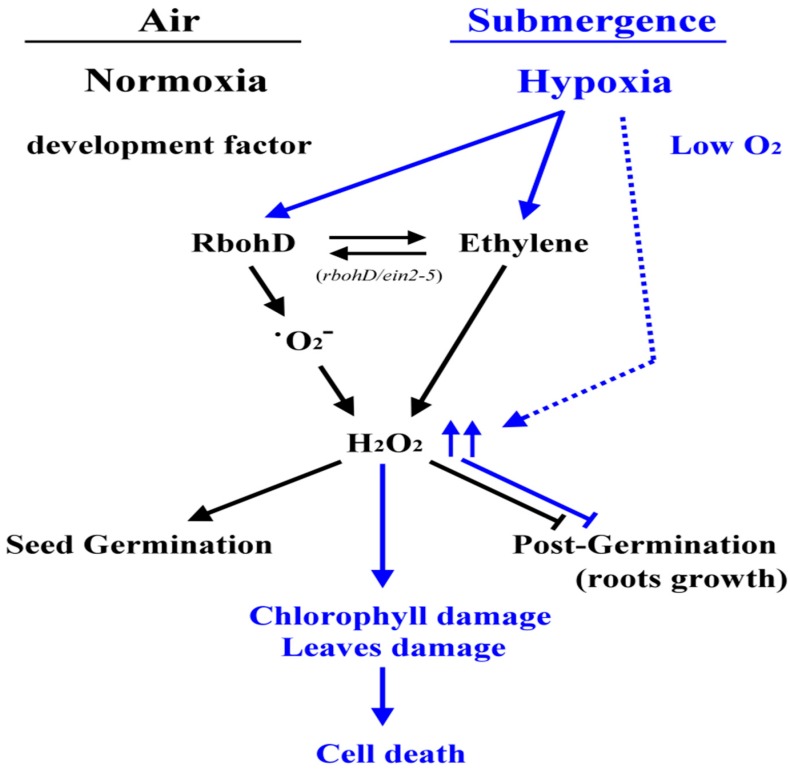
Schematic of the effects of RbohD-derived H_2_O_2_ and ethylene signaling under normoxic and hypoxic conditions. Both RbohD and EIN2 had a synergistic effect on seed germination and root growth at the postgermination stage under normoxic conditions (black line). Hypoxia triggers complex signaling pathways to increase the production of H_2_O_2,_ leading to chlorophyll and leaves damages influencing submergence tolerance in *Arabidopsis* (blue line).

Chlorophyll absorbs the wavelengths associated with violet-blue and orange-red light within the visible light spectrum that are used in photosynthesis during plant growth and development. When plants are exposed to abiotic and biotic stresses such as drought, extreme temperature, cold, or heavy metals, chlorophyll concentration is decreased and protein degradation is increased [41,42,43]. Many studies have reported that ethylene is involved in the regulation of chlorophyll degradation during development or under stress conditions. When plants are submerged, chloroplasts disintegrate and photosynthetic capacity is lost [44,45,46]. In the present study, chlorophyll a and total chlorophyll concentration decreased significantly in *rbohD/ein2-5* double mutants after submergence treatment (Figure 5). Furthermore, the seedlings of the *rbohD/ein2-5* double mutant line had more leaf damage compared with wild-type plants after submergence stress (Figure 6). Some APETALA2/ethylene response factors function in an ethylene-controlled signal transduction pathway to regulate tolerance to hypoxia stress [47,48]. The secondary messenger, H_2_O_2_ production, has also been implicated in regulating tolerance to oxygen deprivation [18,49]. Our results indicate that under hypoxic stress, the induction of transcript levels of the hypoxia-inducible genes encoding ethylene-responsive factor 73 (ERF73) and alcohol dehydrogenase 1 (ADH1) was increased less in the *rbohD/ein2-5* double mutant line than in the *rbohD* and *ein2-5* single mutants (Figure 7). Notably, increased transcript levels of *AtERF73/HRE1* and *ADH1* were induced in all the lines under hypoxic conditions. This suggests that there are signaling elements other than ethylene and RbohD that also modulate the expression of hypoxia-responsive genes, such as *AtERF73/HRE1* and *ADH1*. Our previous results reveal that the accumulation of H_2_O_2_ was reduced in *rbohD*-ko and *ein2-5* during hypoxic stress [19,26]. In this study, both RbohD and EIN2 had a synergistic effect on seed germination and root growth at the postgermination stage under normoxic conditions and influenced submergence tolerance under oxygen deprivation conditions. Taken together, the results reported here indicate the presence of a synergistic interaction between ethylene and H_2_O_2_ signaling in plant development under normoxic conditions and in response to oxygen deprivation (Figure 8).

## 4. Materials and Methods

### 4.1. Plant Materials and Growth Conditions

Wild-type *Arabidopsis thaliana* (ecotype Col-0) was used in this study. The single *ein2-5* and *rbohD* (*At5g47910*) T-DNA insertion mutants (salk_005253C) were obtained from Dr. L.-C. Wang and the Arabidopsis Biological Resource Center (Ohio State University, Columbus, OH, USA), respectively, with the homozygous T-DNA insertion sites confirmed by RT-PCR before use. Double homozygous mutant lines, *rbohD*/*ein2-5*, were obtained by crossing the two single mutants and then screening the F2 and F3 generation as previously described [26]. Seeds were surface sterilized in 70% ethanol for 2 min and then in a 1% bleach solution for 15 min before being washed with sterilized water three times and incubated at 4  °C in the dark for 3 d. Seeds were sown on plates with 0.7% (*w/v*) agar in 1/2-strength Murashige and Skoog (MS) medium containing 0.5% sucrose at pH 5.7. The plates were transferred to a growth chamber at 22  °C with a long photoperiod regimen of 16 h of light (236 μmoLm^−2^s^−1^)/ and 8 h of darkness.

For the seed germination assay, data were collected from 100 seeds of each genotype over three independent experiments. Germination was considered to have occurred when the radicles were 1 mm long. Germination percentage was recorded every 24 h for 4 d. The number of germinated seeds was expressed as a percentage of the total number of seeds plated for the indicated times. The mean germination time was calculated to assess the time spent to germinate or emerge [50].

### 4.2. Seedling Hypoxia, Submergence, and ACC Treatments

For the hypoxia treatment, 14-day-old seedlings were placed on damp filter paper for a 10 min pretreatment and then onto a floating platform with their roots immersed in 1/2 MS solution. The solution was constantly supplied with 3% O_2_/97% N_2_ gas in the dark to replace the oxygen in the solution. The seedling roots were collected for analysis. Other 14-day-old seedlings were placed in the dark without immersion in 1/2 MS solution as controls. For the submergence treatment, seeds were grown on 1/2-strength MS medium in a tissue cultivation box (L:W:H, 12 cm × 9 cm × 7 cm) for 7 d and then treated with or without 250 mL of sterile deionized water for 12 d. The water was drained out for the the subsequent 5-day recovery period. The root lengths of the seedlings (under normoxic conditions or submerged conditions) were measured. Data were collected from at least 100 seedlings of each genotype in three independent experiments. Photographs were taken at the end of the treatment period. For the ACC treatment, seeds were grown on 1/2-strength MS medium with or without 5 μM ACC in the dark for 4 d. The phenotypes were observed, and photographs were taken at the end of the treatment period.

### 4.3. Plant Chlorophyll Content and Leaf Damage Index Measurements

For chlorophyll content assays, the content was determined according to Wintermans and De Mots (1965) after extraction in ethanol [51]. The 7-day-old seedlings were treated under normoxic conditions or submerged conditions for 12 d. Aboveground tissue (50 mg) was collected and ground in 2 mL of sodium phosphate buffer (50 mM pH 6.8), 40 μL of which was added to 960 mL of 99% ethanol and incubated for 30 min at room temperature in the dark with gentle shaking. After centrifugation at 4 °C for 15 min at 1000× *g*, the absorbance values of the supernatant were measured at 665 and 649 nm with a spectrophotometer (Metertec SP8001) to determine to concentrations of chlorophyll a, b, and total chlorophyll. Data were collected from three independent experiments. For leaf damage index measurement, the 7-day-old seedlings were treated under submerged conditions for 12 d followed by 5 d of recovery. The levels of damage were defined as follows: Index 1 = 100% green leaves, Index 2 = over 50% green leaves, Index 3 = less than 50% green leaves, and Index 4 = 0% green leaves. Data were collected from three independent experiments. Each experiment involved the use of at least 25 seedlings. Photographs were taken at the end of experiment. For the hypoxia treatment, 14-day-old seedlings were placed on a floating platform with their roots immersed in 1/2 MS solution supplemented with 0.5% (*w/v*) Suc at pH 5.7. The solution was constantly supplied with 3% (*v/v*) O_2_ and 97% (*v/v*) N_2_ in the dark for 3 h. The root samples were collected and frozen for analysis.

### 4.4. RNA Extraction and Quantitative RT-PCR (qRT-PCR) Analyses

The 14-day-old seedlings were hypoxia-treated for 3 h. Then, root samples were collected in liquid nitrogen and stored at −80 °C until use. Total RNA was extracted using TRIzol (Invitrogen, Carlsbad, CA, USA) and then subjected to DNase treatment using the TURBO DNA-free kit (Ambion, Austin, TX, USA). RNA concentrations were determined, and samples were then reverse-transcribed into cDNAs by using Moloney murine leukemia virus reverse transcriptase (Invitrogen). qRT-PCR was conducted as previously described [52] and performed using a Rotor-Gene 3000 instrument (Corbett Research, Sydney, Australia) with Power SYBR Green PCR master mix (GeneMark, Taipei, Taiwan) in accordance with the manufacturers’ recommendations. The Actin gene was used as an internal control for normalization. Relative expression levels were analyzed with Rotor-Gene 6 software (Corbett). Experiments were repeated five times independently with duplicate samples. The sequences of primers used for qRT-PCR are presented in Table 1.

## 5. Conclusions

In our previous studies, the data indicated that an increase in the transcript levels of *RbohD* was induced at a relatively early stage during hypoxic stress; for other hypoxia-inducible RBOHs, such as *RbohA*, *B*, *F*, *G*, and *I*, transcript expressions were also affected by oxygen deprivation but not at a relatively early stage [24]. The involvement of other signaling elements in the complex signaling network associated with oxygen deprivation requires further evaluation.

## Figures and Tables

**Table 1:** Primers used for quantitative RT-PCR experiments.

Gene Name	Primer Sequence
*rAtERF73/HRE1*-forward	5′-atcatgggcgatgcgaataa-3′
*rAtERF73/HRE1*-reverse	5′-gcgagaaatattcggtctggtt-3′
*rADH1*-forward	5′-catgaacaaggagctggagcttg-3′
*rADH1*-reverse	5′-ctctcccttcagcatgtaatcaaagg-3′
*rRbohD*-forward	5′-ccgagcagacggaggagat-3′
*rRbohD*-reverse	5′-tggaccgtcgataaggacctt-3′
